# P-1228. Posaconazole Oral Suspension vs. Crushed Delayed-Release Tablets: Probability of Pharmacokinetic Target Attainment with Enteral Tube Administration

**DOI:** 10.1093/ofid/ofaf695.1420

**Published:** 2026-01-11

**Authors:** Nirjan Bhattarai, Patrick M Wieruszewski, Dan Ilges, Casey O’Connell, John Robinson, Kristin Cole, Tanner Johnson, Ryan W W Stevens

**Affiliations:** Mayo Clinic, Rochester, MN; Mayo Clinic, Rochester, MN; Mayo Clinic Arizona, Phoenix, Arizona; Mayo Clinic, Rochester, Rosemount, Minnesota; Mayo Clinic Arizona, Phoenix, Arizona; Mayo Clinic, Rochester, MN; Mayo Clinic, Rochester, MN; Mayo Clinic, Rochester, MN

## Abstract

**Background:**

Posaconazole immediate release (IR) suspension is the standard product for enteral feeding tube (EFT) administration but has erratic pharmacokinetic behavior and results in sub satisfactory concentrations. Limited evidence suggests that the posaconazole oral delayed-release (DR) tablets may be crushed.

**Figure:**
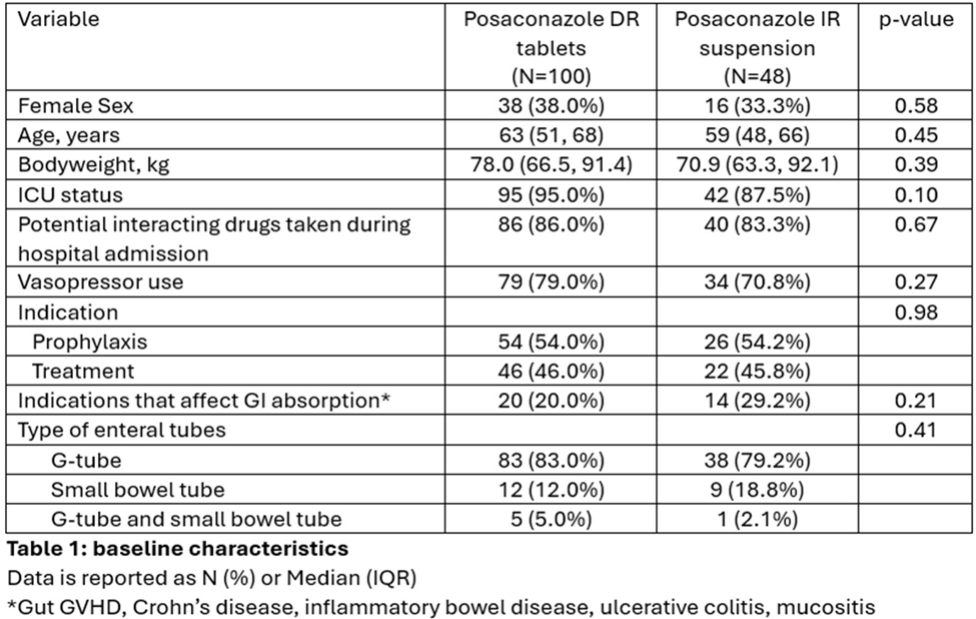

**Figure:**
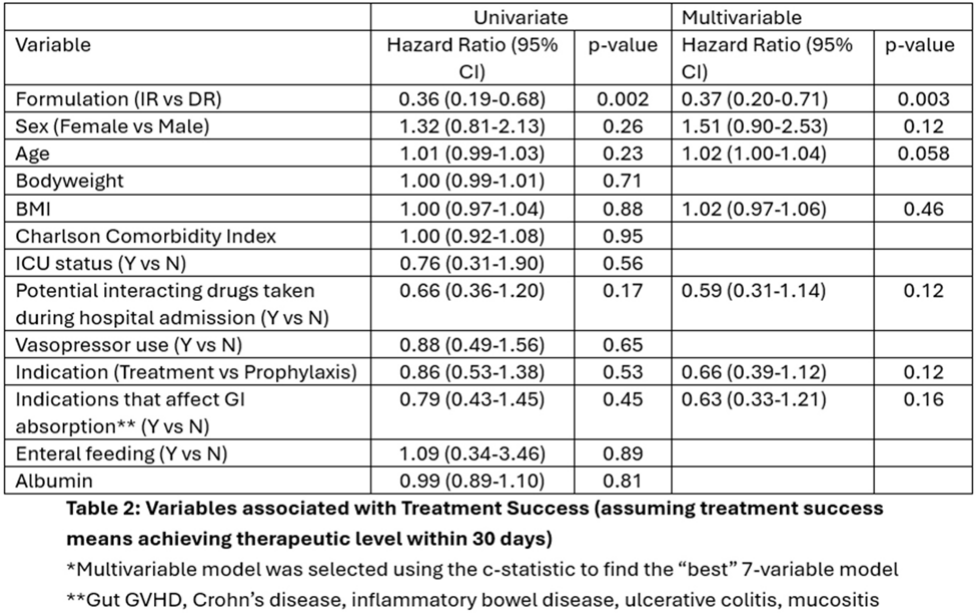

**Methods:**

This was a retrospective cohort study of hospitalized adults who received EFT administration of posaconazole IR suspension or crushed DR tablets between 2018 and 2024. Patients were eligible if they had at least one serum concentration drawn ≥5 days after posaconazole initiation. The primary outcome was the attainment of therapeutic (≥700ng/mL for prophylaxis, ≥1,000ng/mL for treatment) serum concentration within 30 days. Secondary outcomes included time to therapeutic serum concentration, adverse drug effects, and frequency of clogged EFT. Multivariable cox proportional hazards regression was used to assess the relationship between posaconazole formulation and attainment of therapeutic concentrations while adjusting for confounding variables.

**Results:**

Of the included patients, 100 received DR tablets and 48 received IR suspension. There were no significant differences in baseline characteristics or severity of illness. Among DR tablet and IR suspension recipients, 59% and 23% achieved therapeutic concentrations, respectively (p < 0.001). After adjusting for age, sex, BMI, drug interactions, posaconazole indication, malabsorptive gastrointestinal conditions, IR suspension was significantly less likely than crushed DR tablets to achieve therapeutic posaconazole concentrations within 30-days (HR 0.37, 95% CI 0.20-0.71; p < 0.003). The crushed DR tablet group required less total daily posaconazole to achieve therapeutic concentrations (400 vs 600 mg/day, p = 0.003). Clogged EFTs were more frequent in the crushed DR tablet group (27% vs 10%, p = 0.022), while no differences were observed in the occurrence of hepatotoxicity or hypokalemia.

**Conclusion:**

Administration of crushed posaconazole DR tablets via EFT is more likely to attain therapeutic concentrations compared to IR suspension. The optimal technique for administering crushed DR tablets to prevent EFT complications warrants investigation.

**Disclosures:**

Patrick M. Wieruszewski, PharmD, FCCM, Wolters Kluwer/UpToDate: Advisor/Consultant

